# COVID-19 Response Roles among CDC International Public Health Emergency Management Fellowship Graduates

**DOI:** 10.3201/eid2813.220713

**Published:** 2022-12

**Authors:** Sharanya Krishnan, Catherine Espinosa, Michelle N. Podgornik, Seghen Haile, José J. Aponte, Christopher K. Brown, Sara J. Vagi

**Affiliations:** Centers for Disease Control and Prevention, Atlanta, Georgia, USA (S. Krishnan, C. Espinosa, M.N. Podgornik, S. Haile, J.J. Aponte, C.K. Brown, S.J. Vagi);; US Public Health Service Commissioned Corps, Atlanta (S. Krishnan, S.J. Vagi)

**Keywords:** COVID-19, SARS-CoV-2, global public health, emergency management, emergency response, capacity development, global health security agenda, workforce development, respiratory infections, zoonoses

## Abstract

Since 2013, the US Centers for Disease Control and Prevention has offered the Public Health Emergency Management Fellowship to health professionals from around the world. The goal of this program is to build an international workforce to establish public health emergency management programs and operations centers in participating countries. In March 2021, all 141 graduates of the fellowship program were invited to complete a web survey designed to examine their job roles and functions, assess their contributions to their country’s COVID-19 response, and identify needs for technical assistance to strengthen national preparedness and response systems. Of 141 fellows, 89 successfully completed the survey. Findings showed that fellowship graduates served key roles in COVID-19 response in many countries, used skills they gained from the fellowship, and desired continuing engagement between the Centers for Disease Control and Prevention and fellowship alumni to strengthen the community of practice for international public health emergency management.

Public health emergencies originating from outbreaks of emerging infectious diseases have fueled the need for countries to develop their capacities to prevent, detect, and respond to public health threats (*1*–*6*). To manage responses to these threats, countries around the world, beginning in about 2012, began to establish public health emergency operations centers (PHEOCs) (*7*). PHEOCs serve as command centers for coordinating various functions of health emergency responses, such as information management, risk communications, logistics, and operations (*7**,**8*). Establishing PHEOCs introduced the need for trained personnel to manage and operate these facilities. In 2013, the US Centers for Disease Control and Prevention (CDC) responded to this need by establishing the Public Health Emergency Management (PHEM) Fellowship program (https://www.cdc.gov/cpr/eoc/EmergencyManagementFellowship.htm) to help build a workforce to strengthen emergency management capacity among international public health communities (*9*–*11*). The PHEM Fellowship program trains international midcareer public health professionals in emergency management principles using a competency-based curriculum that incorporates lectures, case studies, and participation in real-world experiences. As of 2020, CDC had trained 141 fellows, representing 36 countries worldwide, in 12 semiannual cohorts conducted during August 2013–May 2020. 

The magnitude of the COVID-19 pandemic highlighted the need in many countries for experts with public health emergency management skills to organize, lead, and streamline response efforts. Although anecdotes from the field demonstrated notable roles by PHEM Fellowship program graduates in providing emergency management leadership in COVID-19 response efforts, details of their roles and skills had not been systematically captured. Also, although some fellowship alumni have requested remote technical guidance during the COVID-19 pandemic, the type and extent of technical assistance needed had not been systematically surveyed. Identifying COVID-19 response roles and remaining training needs of PHEM Fellowship graduates can inform curriculum development for future training activities. 

To quantify program graduates’ contributions during COVID-19 and training and assistance needs, CDC staff, in 2021, designed and administered a survey. The objectives of the survey were to assess the number and proportion of total graduates engaged in COVID-19 response in the public health emergency management system in the respondent’s country; identify the organizations served and positions filled by graduates within the country’s public health emergency management system before and during the COVID-19 response; identify public health emergency response skills acquired through the PHEM Fellowship program that the respondent considered useful after graduation; identify additional technical skills related to public health emergency management needed to sustain the COVID-19 response; and identify modes of technical assistance support (remote or onsite) preferred by graduates. 

## Methods

We developed a 21-question web-based survey that could be answered in 10–15 minutes designed to address the 5 survey objectives (Appendix). We analyzed responses grouped by semiannual cohort (n = 12) and region. On March 20, 2021, we sent an email with a secure link to the survey to all persons who had graduated from the PHEM Fellowship program by that date (n = 141). The survey remained open for 5 weeks; reminder emails were sent 2 and 4 weeks after the initial mailing. CDC reviewed the activity and determined that it did not involve human subject research and therefore did not require Institutional Review Board approval. 

The survey collected deidentified information on respondents’ countries, roles, graduation month and year (cohort), and organization type of current and any previous employment. Survey questions required multiple-choice, multiple-answer, free text, or 5-point Likert scale responses (*12*). We created the survey in the Epi-Info Secure Web Survey tool (https://www.cdc.gov/epiinfo/index.html) and included relevant skip patterns to simplify entry for respondents. We used the number of graduates of the PHEM Fellowship program from each country and cohort to determine the expected number of responses, then compared those to survey responses to identify and remove duplicates. 

Once the survey closed, we combined French and English responses based on common data elements, then cleaned and analyzed the data using Epi Info and Microsoft Power BI (https://powerbi.microsoft.com). We calculated response rates using standard definitions (*13*). We calculated 95% CIs around percentages with the finite population correction factor for known population size (n = 141 graduates). To examine differences by region, we organized respondents by their corresponding World Health Organization (WHO) regional offices (Table 1).

**Table 1:** Number of graduates from the US CDC Public Health Emergency Management Fellowship program during 2013–2020 and participants in April 2021 survey of COVID-19 and other activities, by WHO Regional Office

WHO Regional Office	Countries	No. (%) fellowship graduates	No. (%) survey respondents
Africa	African Union, Benin, Burkina Faso, Cameroon, Côte d’Ivoire, Democratic Republic of the Congo, Ethiopia, Ghana, Guinea, Guinea-Bissau, Kenya, Liberia, Mali, Mauritania, Nigeria, Rwanda, Senegal, Sierra Leone, Tanzania, Togo, Uganda	91 (64.5)	62 (69.7)
Eastern Mediterranean	Jordan, Pakistan, Saudi Arabia	6 (4.3)	5 (5.6)
Europe	Kazakhstan, Republic of Georgia	3 (2.1)	2 (2.2)
South-East Asia	Bangladesh, India, Indonesia, Myanmar, Thailand	16 (11.3)	11 (12.4)
Western Pacific	Australia, China, Japan, Malaysia, South Korea, Vietnam	25 (17.7)	9 (10.1)
Total		141 (100)	89 (100)

*CDC, Centers for Disease Control and Prevention; WHO, World Health Organization.

## Results 

We successfully reached 136/141 (96.5%) PHEM Fellowship program graduates. Overall, respondents submitted 111 completed surveys; 21 were duplicates, and 1 did not include country name and cohort (Figure). Duplicates were most likely to occur right after the respondent’s first submission or shortly after reminder emails were distributed. After excluding duplicates and the 1 incomplete response, we analyzed data from the remaining 89 surveys, a response rate of 74.2% (*13*). Respondents from WPRO (Western Pacific Regional Office) countries had a 42.9% response rate, lower than those for other WHO regions: AFRO (Africa), 77.5%; SEARO (South-East Asia), 91.7%; EMRO (Eastern Mediterranean), 100%; and EURO (Europe), 100%. There were no PHEM Fellowship program graduates from the Americas at the time the survey was conducted. Overall, the first 2 cohorts had lower response rates (0% for cohort 1 and 14.3% for cohort 2) and cohort 10 had a higher response rate (93.8%) than those for the other groups combined. The distribution of survey participants by WHO region was similar to the overall distribution of total fellowship participants by region (Table 1). We analyzed the survey data to assess the 5 predetermined survey objectives. 

**Figure:**
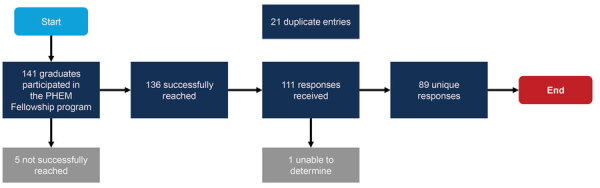
Flowchart illustrating overall summary of survey responses from graduates of the Centers for Disease Control and Prevention PHEM Fellowship program contacted during April 2021. A total of 141 fellows representing 36 countries worldwide have completed the program in 12 semiannual cohorts conducted during August 2013–May 2020. PHEM, Public Health Emergency Management.

### Objective 1: Assess Number and Proportion of Graduates Engaged in the Country’s PHEM System during the COVID-19 Response 

Overall, 80/89 (89.9%) survey respondents have supported their country’s COVID-19 response in various PHEM roles. At the time of the survey, 49/89 (55.1%) respondents had spent 76%–100% of their work time on emergency preparedness or response activities for COVID-19 or any other public health emergency; 17 (19.1%) had spent 51%–75%, 10 (11.2%) had spent 26%–50%, 6 (6.7%) had spent 1%–25%, and 1 (1.1%) had spent no time on these activities. Of the 62 respondents from AFRO, 35 (56.5%) had spent 76%–100% of their time working on emergency management preparedness or response activities, compared with 2/5 (40.0%) EMRO respondents, 4/11 (36.4%) SEARO respondents, and 7/9 (77.8%) WPRO respondents. 

### Objective 2: Identify Organizations and Positions of Graduates in the Country’s Emergency Management System before the PHEM Fellowship and during the COVID-19 Response

The 89 respondents reported diverse professional backgrounds, and many had served in several different positions before participating in the PHEM Fellowship program. More respondents (n = 60, 67.4%) worked for the country’s ministry of health than any other organization type. During the COVID-19 pandemic, more respondents reported engaging in emergency response functions after graduating from the fellowship program than before. More than half (n = 47, 52.8%) served in managerial or nonmanagerial roles in emergency operations centers to support COVID-19 and other public health responses (Table 2). Among respondents, 80/89 (89.9%) supported the COVID-19 response in a variety of traditional incident management system (IMS) functional roles as incident managers or in operations, planning, or logistics support (Table 3). Most respondents reported being involved in COVID-19–related scientific technical assistance (n = 38, 47.5%) or planning (n = 34, 42.5%). 

**Table 2:** Organization, position types, and roles held by Public Health Emergency Management fellowship program graduate survey respondents at time of survey (April 2021) and during the COVID-19 response (January 2020–April 2021)*

Characteristics	No. responses (95% CI)
COVID-19 response organization types, n = 89	
Ministry of health	58 (52.2–64.7)
National public health institute	31 (25.6–37.3)
Other organization	18 (13.1–22.8)
Nongovernmental organization	8 (4.5–11.3)
US Centers for Disease Control and Prevention country office	6 (2.7–8.5)
Local department of health	10 (6.3–13.9)
Animal health sector	6 (2.7–8.5)
Other ministry	8 (4.5–11.3)
World Health Organization	10 (6.3–13.9)
Ministry of defense	1 (–0.2 to 2.5)
Position areas within COVID-19 response organizations, n = 89	
Scientific or technical response	55 (48.8–61.4)
Emergency operations center staff, managerial	53 (46.5–59.1)
Rapid response team manager	35 (28.8–40.9)
Other position	27 (21.3–32.6)
Scientific or technical, nonresponse	26 (20.3–31.4)
Emergency operations center staff, nonmanagerial	18 (13.1–22.8)
COVID-19 roles, global, n = 80†	
Scientific or technical assistance	48 (40.3–54.7)
Planning section	43 (35.3–49.7)
Operations section	35 (28.1–41.9)
Situational awareness	28 (21.0–34.0)
Emergency operations center manager	18 (12.0–23.0)
Rapid response team	18 (12.0–23.0)
Other role	14 (8.8–18.7)
Incident manager	13 (7.7–17.3)
Liaison officer	9 (4.7–12.8)
Logistics section	6 (2.7–9.8)
Public information officer	4 (1.0–6.5)
Finance and administration section	3 (0.2–4.8)
Safety officer	3 (0.2–4.8)

*Respondents could select multiple options so no. of responses can exceed n values. †Three respondents did not support COVID-19 response; 6 respondents did not provide a response to this survey question.

**Table 3:** Confidence to perform emergency management skills acquired in the CDC PHEM Fellowship program as reported by respondents to a survey of program graduates, April 2021*

Skill	Emergency management skills used†				Confidence to perform activity without CDC support‡		
	Agree or strongly agree	Total no.§	% (95% CI)		Confident or very confident	Total no.§	% (95% CI)
IMS, coordination, communication							
Develop a situation report	64	64	100 (100.0–100.0)		55	64	86 (79.6–92.3)
Develop an incident action plan	66	68	97 (94.2–100.0)		55	68	81 (74.1–87.6)
Develop response objectives	72	72	100 (100.0–100.0)		61	72	85 (78.9–90.6)
Develop risk communications	43	45	96 (90.6–100.5)		31	45	69 (57.7–80.1)
Manage meetings	73	75	97 (94.8–99.8)		61	75	81 (75.3–87.4)
Serve in an IMS functional role	66	67	99 (96.4–100.6)		51	67	76 (68.7- 83.5)
Track tasks	61	66	92 (87.8– 97.1)		51	66	77 (69.9–84.7)
Policies, plans, SOPs							
Create a PHEOC handbook	43	46	94 (87.6–99.4)		31	46	67 (56.2–78.6)
Create standard operating procedures	70	70	100 (100.0–100.0)		54	70	77 (70.1–84.1)
Develop a concept of operations	47	50	94 (88.7–99.3)		34	50	68 (57.6–78.4)
Develop an all-hazards plan	44	45	98 (94.2–101.3)		32	45	71 (60.1–82.1)
Develop hazard-specific contingency plans	53	57	93 (87.8–98.1)		38	57	67 (57.2–76.1)
Develop legal authorities for PHEOC	46	48	96 (91.2–100.4)		30	48	63 (51.3–73.7)
Preparedness, exercises, evaluation							
Conduct a risk assessment	50	52	96 (92.0–100.3)		35	52	67 (57.1–77.5)
Conduct an after-action review	54	56	96 (92.6–100.2)		41	56	73 (64.2–82.3)
Contribute to exercise development	60	61	98 (96.0–100.8)		45	61	74 (65.4–82.1)
Facilitate PHEM trainings in-country	65	67	97 (94.1–100.0)		50	67	75 (67.1–82.2)
Perform watch desk duties	48	48	100 (100.0–100.0)		39	48	81 (72.3–90.2)
Other	14	15	93 (81.4–105.3)		10	15	67 (44.0–89.3)

*CDC, Centers for Disease Control and Prevention; PHEM, Public Health Emergency Management; PHEOC, public health emergency operations center; SOP, standard operating procedure. †Complete survey question: The PHEM fellowship program introduces many emergency management skills. Which of those skills have you performed individually or as part of a group, before, during, or after a public health response? ‡Complete survey question: How confident were you in your abilities to implement the activity in your country without any technical support from CDC? §No. respondents answering questions. Questions were dependent on a skip pattern, so no. respondents differed for each question.

### Objective 3: Identify Public Health Emergency Response Skills Acquired through the PHEM Fellowship that Have Been Useful after Graduation

Among respondents, 81/89 (91.0%) indicated they had used >1 specific emergency management skill in the context of COVID-19 and other public health emergencies since graduating from the fellowship program. Among respondents, 97.4% agreed that the fellowship program had provided useful training skills in IMS, coordination, and communication; 96.5% in developing policies, plans, and procedures; 97.5% in preparedness, exercises, and evaluation; and 93.3% in other emergency management skills. Of 19 public health emergency management skills detailed in the survey, respondents reported performing a median of 13 (interquartile range 9–18); 16 respondents reported performing 18/19 skills. When asked about their confidence in performing those skills, 79.9% felt confident performing tasks associated with IMS, coordination, and communication; 69.3% felt confident developing emergency response policies, plans, and procedures; and 73.9% felt confident in their skills for preparedness, exercises, and evaluation (Table 3). 

### Objective 4: Identify Current Technical Needs Related to Emergency Management that Are Critical to the COVID-19 Response

To address COVID-19 response needs, 65/89 respondents (73.0%) requested >1 type of technical support from CDC emergency management specialists. Among the 65 respondents that requested emergency management technical support, 57 (87.7%) requested general support for workforce development in their country, 35 (53.8%) technical support to develop plans and standard operating procedures, 30 (46.2%) support on the PHEOC physical infrastructure, 31 (47.6%) technical support on rapid-response training, and 31 (47.7%) technical support on exercises related to developing, executing, and evaluating responses. 

### Objective 5: Identify Methods of Technical Assistance Desired by Graduates

Among respondents from all cohorts and regions, 73/89 (82.0%) said they would participate in additional PHEM training opportunities, among whom 49/73 (67.1%) preferred a combination of in-person and virtual training modalities, 17 (23.3%) preferred in-person training, 6 (8.2%) virtual training; 1 person (1.4%) did not answer that question. Capacity development support between graduates was common. Among respondents from all cohorts and regions, 55 (61.8%) indicated they had either provided support to or received support from other PHEM fellowship program graduates, 18 (20.2%) had not given or received support, and 16 (18.0%) did not answer that question; 63 respondents (70.8%) said they would be willing to present in future PHEM trainings. 

## Discussion

This survey provided information on how participation in the PHEM Fellowship program contributed to improving international workforce capacity to manage public health emergencies. The PHEM Fellowship program provides standard training and mentorship and networking components that enable countries to build systems unique to their needs and context. Nearly 90% of respondents indicated that they held a role in their country’s COVID-19 response, demonstrating the relevance of a trained public health emergency management workforce during emergencies. Program graduates credited the fellowship with developing skills essential for public health emergency management, including conducting risk assessments, developing response plans, aiding with training and exercises, and managing resources, and most expressed interest in sharing their experiences.

PHEM fellowship program graduates are likely more culturally aware of local politics, resources, languages, and challenges than are US-based experts. Anecdotal examples from the survey of initiatives by graduates in the field included conducting reciprocal site visits between Uganda and Sierra Leone to observe how other nations operate their PHEOCs and providing technical assistance (e.g., Cameroon supporting the Democratic Republic of the Congo by sharing risk assessment and response plans). Other opportunities for in-depth, longitudinal relationship management with PHEM graduates are likely and could be modeled after the alumni engagement networks of other CDC training programs. 

All data were self-reported and therefore possibly subject to biases that tend toward overestimates. Respondents might have been more likely to rate their skills and confidence more positively (social desirability bias) and attribute skills gained from a CDC program more highly on a survey conducted by CDC (acquiescence bias) (*14*). Several factors might have suppressed the overall response rate, including differences in language understanding and perception, length of time since participation in the fellowship, lack of time because of engagement in the COVID-19 response, or incorrect contact information. Regions such as WPRO were underrepresented in survey responses compared with AFRO, which could affect generalizability of the results and subsequent programmatic recommendations. 

Overall, our findings indicated that fellowship graduates served key roles in country COVID-19 responses, used skills gained from participating in the fellowship, and desired ongoing engagement between CDC and fellowship alumni to continue strengthening the community of practice for international public health emergency management. Investments in this program could address the growing demand for public health emergency responders with the expertise to combat future epidemics and pandemics (*15**,**16*). Response needs prompted by the COVID-19 pandemic have increased interest from more countries and regions to provide applicants to future fellowship cohorts (CDC PHEM Fellowship Program, unpub. data). 

Strong investments in building international workforce capacity should combine time-limited intensive in-person learning with ongoing mentorship and cultivated alumni networks. CDC is working to expand the fellowship’s curriculum, develop advanced training opportunities, and translate materials into additional languages. The goal of these training improvements and advancement of peer-to-peer mentoring is to continue strengthening international public health emergency management workforce capacity. Using a combination of virtual and in-person trainings, peer-to-peer learning, and sharing best practices can strengthen the nascent global network of fellowship graduates and other public health emergency management experts. As the field of public health emergency management continues to advance, systematic evaluations are needed to understand how best to support PHEM fellowship program graduates and identify strengths and gaps of the program at large. CDC is developing an evaluation framework and evaluation plan to address this need. 

Appendix Survey questions from Assessment of Public Health Emergency Management Fellowship graduates.
