# Supplementary material for: COVID-19 Response Roles among CDC International Public Health Emergency Management Fellowship Graduates
Source: Emerg Infect Dis. 2022 Dec;28(Suppl 1):S145–50. doi: 10.3201/eid2813.220713 (PMC9745245; doi:10.3201/eid2813.220713)
Supplement: Appendix — Survey questions from Assessment of Public Health Emergency Management Fellowship graduates. [file 22-0713-Techapp-s1.pdf]

Article DOI: https://doi.org/10.3201/eid2813.220713

COVID-19 Response Roles among International Public Health Emergency Management Fellowship Graduates

Appendix

Assessment of Public Health Emergency Management Fellowship Graduates

Survey Questions

(Presurvey Questions)

1. Please indicate your preferred language/indiquez votre langue préférée, s'il vous plaît.
   - o English/Anglais
   - o French/Français

The goal of this survey is to learn about your experience after you participated in the Public Health Emergency Management (PHEM) Fellowship program at the Centers for Disease Control and Prevention (CDC). We would like to know about:

- How the PHEM Fellowship can be improved
- How attending the PHEM Fellowship contributes to PHEM program establishment and maintenance in your country
- Technical support that may strengthen the implementation of the PHEM program in your country
- Your involvement in COVID-19 response efforts in your country

Additionally, we are seeking to gauge interest in future PHEM training opportunities for PHEM Fellowship graduates.

The survey will take about 15 minutes to complete. Your answers will be kept private. The questions that ask about the country in which you work and your PHEM graduation date will only be used to describe your responses to this survey in aggregate with other respondents. No personal names or country names will be reported.

Lastly, completing the survey is voluntary. Try to answer all the questions. There are no wrong answers. Neither you nor your country will be penalized in any way if you choose not to answer some or all the questions.

2. Do you consent to take this survey?
   - o Yes
   - o No (Stop and exit the survey)

(Survey Questions)

1. Please indicate the country you represented at the time of the PHEM Fellowship.
   - *Dropdown list of countries that participated in the PHEM Fellowship program*
2. When did you graduate from the PHEM Fellowship program?
   - *Dropdown list of month and year of graduation*
3. Select all organizations for which you worked **immediately before entering** the PHEM Fellowship. (Select all that apply)
   - ☐ Ministry of Health
   - ☐ Ministry of Defense
   - ☐ Animal Health Sector
   - ☐ National Ministry other than Ministry of Health or Ministry of Defense (Please specify)
   - ☐ National Public Health Institute
   - ☐ Local Department of Health
   - ☐ World Health Organization
   - ☐ Centers for Disease Control and Prevention Country Office
   - ☐ Non-Governmental Organization
   - ☐ Other (Please specify)
   - ☐ Other (Please specify)
   - ☐ Other (Please specify)
4. For each organization selected in question 3, select all positions you held at that organization. (Select all that apply) [Pose this question for each organization selected in Q3.]
   - ☐ Permanent Emergency Operations Center (EOC) staff (managerial)
   - ☐ Permanent EOC staff (non-managerial)
   - ☐ Rapid Response Team (RRT) manager
   - ☐ Response-related scientific/technical position (e.g., Surveillance/Epidemiology, Laboratory, Infection Prevention and Control (IPC), Border Health, etc.)
   - ☐ Nonresponse-related scientific/technical position (e.g., Surveillance/Epidemiology, Laboratory, IPC, Border Health, etc.)
   - ☐ Other (Please specify)
   - ☐ Other (Please specify)
   - ☐ Other (Please specify)
5. Select all organizations for which you currently work. (Select all that apply)
   - ☐ Ministry of Health
   - ☐ Ministry of Defense
   - ☐ Animal Health Sector
   - ☐ National Ministry other than Ministry of Health or Ministry of Defense (Please specify)
   - ☐ National Public Health Institute
   - ☐ Local Department of Health
   - ☐ World Health Organization
   - ☐ Centers for Disease Control and Prevention Country Office
   - ☐ Non-Governmental Organization
   - ☐ Other (Please specify)
   - ☐ Other (Please specify)

☐ Other (Please specify)

6. For each organization selected in question 5, select all positions you currently hold at that organization (Select all that apply) [Pose this question for each organization selected in Q5.]
   ☐ Permanent EOC staff (managerial)
   ☐ Permanent EOC staff (non-managerial)
   ☐ RRT manager
   ☐ Response-related scientific/technical position (e.g., Surveillance/Epidemiology, Laboratory, IPC, Border Health, etc.)
   ☐ Nonresponse-related scientific/technical position (e.g., Surveillance/Epidemiology, Laboratory, IPC, Border Health, etc.)
   ☐ Other (Please specify)
   ☐ Other (Please specify)
   ☐ Other (Please specify)
7. What percentage of your combined time is currently spent working on emergency management preparedness and/or response activities (including COVID-19 or any other public health emergency)?
   ○ 0
   ○ 1–25%
   ○ 2–50%
   ○ 51–75%
   ○ 76–100%
8. Have you ever worked or are you currently working on the COVID-19 response in your country?
   ○ Yes
   ○ No
9. (If "Yes" to Q8) In what capacity were you or are you involved in the COVID-19 response? (Select <u>all</u> that apply)
   ☐ EOC Manager
   ☐ Incident Manager
   ☐ Situational Awareness
   ☐ Operations Section
   ☐ Planning Section
   ☐ Logistics Section
   ☐ Finance and Administration Section
   ☐ Public Information Officer
   ☐ Risk Communications
   ☐ Liaison Officer
   ☐ Safety Officer
   ☐ RRT Manager
   ☐ Scientific technical assistance (e.g. Surveillance/Epidemiology, Laboratory, IPC, Border Health, etc.)
   ☐ Other (Please specify)
10. Have you or your organization requested emergency management technical support from CDC for the **COVID-19 response**?
    ○ Yes
    ○ No

11. (If “Yes” to Q10) Which of the following areas of technical support did you or your organization request? (Select all that apply) [Put Q11 on its own page. Definitions for each response option will appear in a box located after the question prompt and its responses.]
    - ☐ EOC physical infrastructure
    - ☐ Plans, standard operating procedures (SOPs), protocols, guidelines, and/or policies
    - ☐ Public health law
    - ☐ Rapid Response Team
    - ☐ Medical countermeasures
    - ☐ Crisis and Emergency Risk Communication (CERC)
    - ☐ Training
    - ☐ Exercises
    - ☐ Establishing an in-country PHEM Fellowship Program
    - ☐ Other (Please specify)

EOC physical infrastructure	Technical support including but not limited to EOC facility design, information technology (IT) and information security, telecommunication equipment and services, and COOP site development.
Plans, standard operating procedures (SOP), protocols, guidelines, and/or policies	Technical support for the development of plans, SOPs, and other PHEOC documentation.
Public health law	Technical support for the development of legislation or an executive directive for the health ministry or public health agency to establish and manage a PHEOC.
Rapid Response Team	Technical support regarding the PHEOC’s identification and deployment of a roster of trained personnel, equipment, and supplies to field sites in the event of a public health emergency.
Medical countermeasures	Technical support on developing plans and/or exercises to validate plans involving distribution of medical countermeasures (life-saving medication, medical supplies etc.)
Crisis and Emergency Risk Communication (CERC)	Technical support on developing an evidence-based framework and best practices for anyone who communicates on behalf of an organization responding to a public health emergency.
Training	Technical support on training workforce in country or developing an in-country training program on emergency management topics (e.g., EOC management and operations, IMS section roles and responsibilities, contingency planning, THIRA, exercise design).
Exercises	Technical support on developing, executing, and evaluating discussion- or operations-based exercises.

12. Since completing the PHEM Fellowship, have you or your organization requested emergency management technical support from CDC for **any other public health response** (e.g., infectious disease outbreak, natural disasters, mass gatherings, man-made disaster)?
    - o Yes
    - o No

13. (If "Yes" to Q12) Which of the following areas of technical support did you or your organization request? (Select all that apply) [Put Q13 on its own page. Definitions for each response option will appear in a box located after the question prompt and its responses.]
   ☐ EOC physical infrastructure
   ☐ Plans, standard operating procedures (SOPs), protocols, guidelines, and/or policies
   ☐ Public health law
   ☐ Rapid Response Team
   ☐ Medical countermeasures
   ☐ Crisis and Emergency Risk Communication (CERC)
   ☐ Training
   ☐ Exercises
   ☐ Establishing an in-country PHEM Fellowship Program
   ☐ Other (Please specify)

EOC physical infrastructure	Technical support including but not limited to EOC facility design, information technology (IT) and information security, telecommunication equipment and services, and COOP site development.
Plans, standard operating procedures (SOP), protocols, guidelines, and/or policies	Technical support for the development of plans, SOPs, and other PHEOC documentation.
Public health law	Technical support for the development of legislation or an executive directive for the health ministry or public health agency to establish and manage a PHEOC.
Rapid Response Team	Technical support regarding the PHEOC's identification and deployment of a roster of trained personnel, equipment, and supplies to field sites in the event of a public health emergency.
Medical countermeasures	Technical support on developing plans and/or exercises to validate plans involving distribution of medical countermeasures (life-saving medication, medical supplies etc.)
Crisis and Emergency Risk Communication (CERC)	Technical support on developing an evidence-based framework and best practices for anyone who communicates on behalf of an organization responding to a public health emergency.
Training	Technical support on training workforce in country or developing an in-country training program on emergency management topics (e.g., EOC management and operations, IMS section roles and responsibilities, contingency planning, THIRA, exercise design).
Exercises	Technical support on developing, executing, and evaluating discussion- or operations-based exercises.

14. The PHEM Fellowship introduces many emergency management skills. Which of those skills have you performed individually or as part of a group, before, during, or after a public health response? (Select all that apply)
   ☐ Developed decree, edicts, or other legal authorities for the PHEOC
   ☐ Conducted a risk assessment (THIRA, STAR etc.)

☐ Developed a Concept of Operations (CONOPS)
☐ Developed All-Hazards Plan
☐ Developed contingency plans for specific threats and hazards
☐ Contributed to the development of a discussion- or operations-based exercise
☐ Created a PHEOC Handbook
☐ Created SOPs that can be utilized during a public health response
☐ Performed duties as part of a "watch desk" or equivalent entity in the PHEOC
☐ Developed a SITREP
☐ Served in any IMS functional role during a response
☐ Developed risk communication messaging using CERC principles
☐ Developed response objectives
☐ Developed an Incident Action Plan
☐ Managed meetings (e.g., set agenda, performed time management tasks, developed purpose, set up room)
☐ Tracked tasks
☐ Conducted an After Action Review and developed a Corrective Action Plan
☐ Facilitated PHEM trainings in your country
☐ Other (Please specify)

15. (For each response selected in Q14) How confident were you in your abilities to implement the activity in your country without any technical support from CDC?
    - Not Confident
    - Somewhat Confident
    - Neutral/Not Applicable
    - Confident
    - Very Confident
16. (For each response selected in Q14) How much do you agree with the following statement? Participation in the PHEM Fellowship provided me with the knowledge and skills to perform this activity in my home country.
    - Strongly Disagree
    - Somewhat Disagree
    - Neutral/Not Applicable
    - Agree
    - Strongly Agree
17. Since graduating from the PHEMF, have you had the opportunity to provide PHEM support to and/or receive PHEM support from graduates in your cohort or a different cohort?
    - Yes (Please elaborate)
    - No

Would you be willing to participate in additional training offered to PHEMF graduates?
- Yes
  - ☐ Virtual
  - ☐ In person
- No

18. (If "Yes, Virtual" to Q18) Please specify what training and/or discussion topics would be most helpful to you to participate in virtually.
19. (If "Yes, In person" to Q18) Please specify what training and/or discussion topics would be most helpful to you to participate in person.

20. Would you be interested and willing to present to PHEMF graduates regarding specific aspects of PHEM implementation in your country?
    - Yes
    - No
